# 

**Published:** 1858-05

**Authors:** 


NORTH AMERICAN

### MEDICO-CHIRURGICAL REVIEW.

Vol. II.] MAY, 1858. [No. 3.

### CONTENTS.

### ANALYTICAL AND CRITICAL REVIEWS.

ART. PAGE

I.—A Manual of Medical Diagnosis. By A. W. BARCLAY, M.D. . . . 401

II.—Handbuch der Speciallen Pathalogie und Therapie. By Prof. R. VIRCHOW . . . . . . . . . . . . 413

III.—The Principles and Practice of Obstetrics. By HENRY MILLER, M.D. . . . . . . . . . . . 436

IV.—The Dispensatory of the United States. By GEORGE B. WOOD, M.D., and FRANKLIN BACHE, M.D. . . . . . . 465

V.—Plates Illustrative of Wilson on Diseases of the Skin . . . . 466

VI.—The Diseases of Children. By FLEETWOOD CHURCHILL, M.D. . 466

### ORIGINAL COMMUNICATIONS.

I.—On the Perceptive Power of the Spinal Cord, as manifested by experiments on Cold-blooded Animals. By GEORGE PATON, M.D. . . . . . . . . . . . . . . . 467

II.—Observations on the Supposed Relations between Epizootics and Epidemics, and the Effects which Diseased Meat may have on Human Health. By GEORGE SUTTON, M.D. . . . . 483

III.—Practical Miscellanies on Diseases of Children. By L. W. MAUTHNER, M.D. . . . . . . . . . . . . 504

IV.—Proceedings of the Pathological Society of Philadelphia . . 506

### AMERICAN PROGRESS OF MEDICAL SCIENCE.

Report on Practical Surgery. By EDWARD HARTSHORNE, M.D., One of the Surgeons of Wills' Hospital, Philadelphia . . . . . . . . 536

### BI-MONTHLY PERISCOPE.

#### PRACTICAL MEDICINE.

1. On Emboli . . . . . . . . . . . . . . . . 568

2. Valerianate of Ammonia successfully used in Neuralgia . . . . 570

PAGE

MATERIA MEDICA AND THERAPEUTICS.

On the Medicinal Action of Lupuline . . . . . . . . . 570

SURGERY.

1. Case of Imperforate Anus . . . . . . . . . . . . 575
2. Large Popliteal Aneurism treated by Pressure, and afterwards by Ligature . . . . . . . . . . . . . 579
3. Malignant Tumor near the Breast at an unusually Early Age . . . 580
4. Excision of the Ankle-Joint . . . . . . . . . 580
5. Collodion and Castor Oil as an Artificial Cuticle . . . . 580

OBSTETRIC MEDICINE.

On Secondary Affections of the Joints in Puerperal Women. . . . 581

EDITORS' TABLE.—Medical Students in the United States during the Session of 1857–58 . . . . . . . . . . 585
Medical Students Abroad . . . . . . . . . . 585
Philadelphia College of Pharmacy . . . . . . . . 585
Resignations and Appointments in Medical Schools . . . . 585
The Medical and Surgical Reporter . . . . . . . . 586
Journal de la Physiologie de l'Homme et des Animaux . . . . 586
American Medical Association . . . . . . . . . 586
American Veterinary Journal . . . . . . . . . 586
The Ecraseur in Ovariotomy . . . . . . . . . 587
Accidents of Knighthood . . . . . . . . . . 587
Appointment of Professor Dickson . . . . . . . . . 588

NECROLOGICAL NOTICES.—Professor J. K. Mitchell . . . . . . 588
Dr. Benjamin Travers . . . . . . . . . . 590
Dr. J. K. Walther . . . . . . . . . . . 591
Dr. Schœpf Merei . . . . . . . . . . . 591
Mons. L. Baudens . . . . . . . . . . . 591
Dr. Forbes Royle . . . . . . . . . . . 591

BI-MONTHLY BIBLIOGRAPHICAL RECORD . . . . . . . . . 592

